# Antibody affinity maturation and cross-variant activity following SARS-CoV-2 mRNA vaccination: Impact of prior exposure and sex

**DOI:** 10.1016/j.ebiom.2021.103748

**Published:** 2021-12-10

**Authors:** Juanjie Tang, Gabrielle Grubbs, Youri Lee, Chang Huang, Supriya Ravichandran, David Forgacs, Hana Golding, Ted M Ross, Surender Khurana

**Affiliations:** a Division of Viral Products, Center for Biologics Evaluation and Research (CBER), FDA, Silver Spring, Maryland, USA, 20993; b Center for Vaccines and Immunology, University of Georgia, Athens, Georgia, USA, 30602; c Department of Infectious Diseases, University of Georgia, Athens, Georgia, USA, 30602

**Keywords:** SARS-CoV-2, COVID-19, Vaccine, Virus neutralization, Affinity maturation, Sex differences

## Abstract

**Background:**

Limited knowledge exists regarding antibody affinity maturation following mRNA vaccination in naïve vs. COVID-19 recovered individuals and potential sex differences.

**Methods:**

We elucidated post-vaccination antibody profiles of 69 naïve and 17 COVID-19 convalescent adults using pseudovirus neutralization assay (PsVNA) covering SARS-CoV-2 WA-1, variants of concern (VOCs) and variants of interest (VOIs). Surface Plasmon Resonance (SPR) was used to measure antibody affinity against prefusion spike and receptor binding domain (RBD) and RBD mutants.

**Findings:**

Higher neutralizing antibodies were observed in convalescent vs. naïve adults against, WA-1, VOCs, and VOIs. Antibody binding to RBD and RBD mutants showed lower binding of post-vaccination sera from naïve compared with convalescent individuals. Moreover, we observed early antibody affinity maturation in convalescent individuals after one vaccine dose and higher antibody affinity after two doses compared with the naïve group. Among the naïve participants, antibody affinity against the SARS-CoV-2 prefusion spike was significantly higher for males than females even though there were no difference in neutralization titers between sexes.

**Interpretation:**

This study demonstrates the impact of prior infection on vaccine-induced antibody affinity maturation and difference in antibody affinity between males and females. Further studies are needed to determine whether antibody affinity may contribute to correlates of protection against SARS-CoV-2 and its variants.

**Funding:**

The antibody characterization work described in this manuscript was supported by FDA's Medical Countermeasures Initiative (MCMi) grant #OCET 2021-1565 to S.K and intramural FDA-CBER COVID-19 supplemental funds. The SPARTA program was supported by the National Institute of Allergy and Infectious Diseases (NIAID), U.S. National Institutes of Health (NIH), Department of Health and Human Services contract 75N93019C00052, and the University of Georgia (US) grant UGA-001. T.M.R is also supported by the Georgia Research Alliance (US) grant GRA-001. The CTRU was supported by the National Center for Advancing Translational Sciences of the National Institutes of Health under Award Number UL1TR002378.

**Research in Context:**

**Evidence before this study**

Long term impact of antibodies elicited by SARS-CoV-2 vaccination is likely to be determined both by the level of virus neutralizing antibody titers, and their affinity. The rapid emergence of SARS-CoV-2 variants around the globe is associated with different levels of resistance to neutralization by post-vaccination sera. Recent reports suggested that immune response elicited by a single dose of vaccine in COVID-19 recovered individuals was comparable with the post-second dose in naïve individuals. However, limited knowledge exists on antibody affinity maturation following first vs second dose in naive vs convalescent individuals and in males *vs* females.

**Added value of this study**

The antibody affinity maturation towards the prefusion spike and RBD was evaluated in SARS-CoV-2 mRNA vaccine recipients that were either naïve or recovered from COVID-19. Post-vaccination antibody affinity was significantly higher for the convalescent individuals compared with naïve group. Higher antibody affinity against the prefusion spike correlated well with virus neutralization with improved titers against multiple SARS-CoV-2 variants. Unexpectedly, among the naïve participants, post-vaccination antibody affinity against the prefusion spike was significantly higher for males than females.

**Implications of all the available evidence**

This study underscores the importance of measuring antibody affinity maturation following vaccination in different target populations (age, sex, immune status) over time as part of the search for correlate of protection against SARS-CoV-2 variants.

Alt-text: Unlabelled box

## 1 Introduction

The rapid emergence of SARS-CoV-2 variants of concern (VOCs) and variants of interest (VOI) around the globe [1] is associated with different levels of resistance to neutralization by convalescent plasma, neutralizing monoclonal antibodies, as well as post vaccination sera [2], [3], [4]. The main VOC/VOIs include the B.1.1.7 (Alpha), the B1.351 (Beta), the P.1 (Gamma), B.1.429 (Epsilon) and B.1.617.2 (Delta) strains [1,[5], [6], [7], [8]]. Another variant that has been identified as a VOIs include the B.1.617.1 (Kappa) strain. Multiple studies are evaluating the effectiveness of SARS-CoV-2 vaccines against circulating SARS-CoV-2 strains and the emerging VOCs/VOIs [9], [10], [11], [12], [13], [14]. Interestingly, several VOC/VOIs share one or more common mutations in the RBD (i.e., N501, K417 and E484) [1] that influence SARS-CoV-2 neutralizing activity against the VOCs [14], [15], [16], [17], [18].

Recent reports suggested that immune response (both B and T cells) elicited by a single dose of vaccine in COVID-19 recovered individuals was comparable with the post-second dose in naïve individuals, and second dose did not have significant impact on the immune response in convalescent individuals [19], [20], [21], [22], [23]. However, limited knowledge exists regarding the impact of SARS-CoV-2 vaccination on the quality of the antibody response in terms of antibody affinity maturation in naive vs convalescent individuals and in males *vs* females.

In the current study, we performed quantitative and qualitative analysis of the vaccine-induced antibody response in naïve vs. previously infected and recovered (aka convalescent) individuals and elucidated potential differences between male and female subjects. Neutralization of SARS-CoV-2 WA-1 strain and several VOCs/VOIs was measured in a pseudovirus neutralization assay (PsVNA) [24]. SPR was used to measure antibody binding to RBD engineered to express key amino acid mutations of the VOCs. Antibody dissociation rates were measured as a surrogate of antibody affinity against SARS-CoV-2 pre-fusion stabilized spike protein and RBD [25], [26], [27], [28], [29].

## 2 Methods

### 2.1 Study design

Heat inactivated de-identified samples were obtained from participants enrolled in the SPARTA (SARS2 Seroprevalence and Respiratory Tract Assessment) program in Athens, GA (USA) with written informed consent (Table S1). The study procedures, informed consent, and data collection documents were reviewed and approved by the WIRB-Copernicus Group Institutional Review Board (WCG IRB #202029060) and the University of Georgia. Samples were tested in different antibody assays with approval from the U.S. Food and Drug Administration's Research Involving Human Subjects Committee (FDA-RIHSC) under exemption protocol ‘252-Determination- CBER-2020-08-19. All samples were tested in duplicates in a blinded fashion. Most absolute values and fold-change graphs were normalized to Log2 for statistical calculations.

### 2.2 Lentivirus pseudovirion neutralization assay

Antibody preparations were evaluated by SARS-CoV-2 pseudovirus 50% neutralization assay (PsVNA50) using WA-1, B.1.1.7, B.1.429, P.1, B.1.351, B.1.617.1 and B.1.617.2 strains (Table S2). The PsVNA using 293-ACE2-TMPRSS2 cell line was described previously [26,30]. Controls included cells only, virus without any antibody and positive sera. The cut-off value or the limit of detection for the neutralization assay is 1:10.

### 2.3 Proteins

The SARS-CoV-2 Spike plasmid expressing genetically stabilized pre-fusion 2019‐nCoV_S‐2P spike ectodomain, gene encoding residues 1-1208 of 2019‐nCoV S fused to 8xHisTag was a kind gift from Barney Graham (VRC, NIH). This expression vector was used to transiently transfect FreeStyle293F cells (ThermoFisher, Catalog number: R79007) using polyethylenimine. Protein was purified from filtered cell supernatants using StrepTactin resin (Cytiva, Catalog number: 29401326) and subjected to additional purification by size‐exclusion chromatography in PBS.

Recombinant SARS-CoV-2 spike RBD and its mutants were purchased from Sino Biologicals (RBD; 40592-V08H, RBD-K417N; 40592-V08H59, RBD-N501Y; 40592-V08H82 and RBD-E484K; 40592-V08H84). Recombinant purified RBD proteins used in the study were produced in 293 mammalian cells. The native receptor-binding activity of the spike RBD proteins was determined by binding to 5 µg/mL of human ACE2 protein [26,29,30].

### 2.4 Antibody binding kinetics of post-SARS-CoV-2 vaccination human serum to recombinant SARS-CoV-2 prefusion spike and RBD proteins by SPR

Steady state equilibrium binding of post-SARS-CoV-2 infected human polyclonal serum was monitored at 25°C using a ProteOn surface plasmon resonance (BioRad). The purified recombinant SARS-CoV-2 proteins were captured to a Ni-NTA sensor chip (BioRad, Catalog number: 176-5031) with 200 resonance units (RU) in the test flow channels. The protein density on the chip was optimized such as to measure monovalent interactions independent of the antibody isotype [27]. Serial dilutions (10-, 50- and 250-fold) of freshly prepared sample in BSA-PBST buffer (PBS pH 7.4 buffer with Tween-20 and BSA) were injected at a flow rate of 50 µl/min (120 sec contact duration) for association, and disassociation was performed over a 600-second interval. Responses from the protein surface were corrected for the response from a mock surface and for responses from a buffer-only injection**.** Total antibody binding was calculated with BioRad ProteOn manager software (version 3.1). All SPR experiments were performed twice., In these optimized SPR conditions, the variation for each sample in duplicate SPR runs was <6%. The maximum resonance units (Max RU) shown for 10-fold diluted serum sample.

Antibody off-rate constants, which describe the stability of the antigen-antibody complex, i.e., the fraction of complexes that decays per second in the dissociation phase, were determined directly from the human polyclonal sample interaction with recombinant purified SARS-CoV-2 prefusion spike ectodomain and RBD using SPR in the dissociation phase only for the sensorgrams with Max RU in the range of 10–150 RU and calculated using the BioRad ProteOn manager software for the heterogeneous sample model as described before [25], [26], [27]. Off-rate constants were determined from two independent SPR runs.

### 2.5 Statistical Analysis

All experimental data to compare differences between groups were analyzed using lme4 and emmeans packages in R (RStudio version 1.1.463).

The initial baseline demographics of these study participants are shown in Table S1. Since age and the body mass index (BMI) can be biologically plausible confounders, data from SPR (antibody binding and antibody off-rates) and neutralization titers (absolute values and fold changes) were analyzed for statistical significance amongst convalescent vs unexposed naïve groups or comparisons of males vs females to control for age and BMI as covariates (predictor variables) using a multivariate linear regression model. To ensure robustness of the results, absolute measurements were log2-transformed before performing the analysis. For comparisons between two vaccine categories (factor variable), pairwise comparisons were extracted using ‘emmeans’ and Tukey-adjusted p values were used for denoting significance to reduce Type 1 error due to multiple testing. The tests were two-sided tests. The statistical analysis tested for significant differences in antibody binding, antibody off-rates, and neutralization titer measurements among naïve vs convalescent vaccine categories and the p-values are shown in Table S3.

Correlation and regression analyses were performed by computing Spearman's rank correlation coefficient and significance in GraphPad Prism.

Power analysis for sample size calculations were performed assuming a power value (beta) as 0.95, 0.9 and 0.8, in the order of decreasing stringency to eliminate Type I error. A significance level of 0.05 was used for sample size calculations. These calculations showed that we needed a sample size of 13, 11 and 8, respectively that are within the actual sample size used in the current study. Hedge's ‘g’ was determined for effect size calculations using the ‘effsize’ package in R. Results for the mean from different comparison groups and an effect size established based on the g values are shown in Table S4.

Experiments were performed based on sample availability during the initial vaccine campaign and hence sample size calculations were not done a priori. However, power analysis calculations were performed to ensure adequate sample sizes (albeit different for naïve, n=69 and COVID-19, n=17 groups) using the 'pwr' package in R. Respective Hedge's g values for those comparisons with a 'large' effect were used for unequal sample size (n). Acceptable values for a sound statistically powered experiment ranged from 0.84-1.

Samples were allocated randomly to each test group and tested in blinded fashion (researcher was blinded to sample identity) to minimize selection bias or detection bias. There were no exclusion criteria. All samples and data were used for analysis and presented in the study.

### 2.6 Data and Materials availability

All data needed to evaluate the conclusions in the paper are present in the paper and/or the Supplementary Materials. The materials generated during the current study are available from the corresponding author under a material transfer agreement on reasonable request.

## 3 Ethics

The study at CBER, FDA, was conducted with de-identified samples and all assays performed fell within the permissible usages in the original consent. Antibody assays were performed with approval from the U.S. Food and Drug Administration's Research Involving Human Subjects Committee (FDA-RIHSC) under exemption protocol ‘252-Determination- CBER-2020-08-19.

## 4 Role of funders

The antibody characterization work described in this manuscript was supported by FDA's MCMi grant #OCET 2021-1565 to S.K and intramural FDA-CBER COVID-19 supplemental funds. The SPARTA program was supported by the National Institute of Allergy and Infectious Diseases (NIAID), U.S. National Institutes of Health (NIH), Department of Health and Human Services contract 75N93019C00052, and the University of Georgia (US) grant UGA-001. T.M.R is also supported by the Georgia Research Alliance (US) grant GRA-001. The CTRU was supported by the National Center for Advancing Translational Sciences of the National Institutes of Health under Award Number UL1TR002378. The funders had no role in study design, data collection and analysis, interpretation, writing, decision to publish, or preparation of the manuscript.

The content of this publication does not necessarily reflect the views or policies of the Department of Health and Human Services, nor does mention of trade names, commercial products, or organizations imply endorsement by the U.S. Government.

## 5 Results

### 5.1 Neutralizing antibody titers of post-vaccination serum from COVID-19 convalescent and naïve adults against various SARS-CoV-2 strains

The objective of this study was to investigate the post-vaccination induced quantitative and qualitative antibody response in seronegative naive males vs females (N=69) compared with adults who were COVID-19 convalescent (N=17) against vaccine-homologous SARS-CoV-2 strain (WA-1) and emerging VOC/VOI strains including Alpha (B.1.1.7), Epsilon (B.1.429), Gamma (P.1), Beta (B.1.351), Kappa (B.1.617.1) and Delta (B.1.617.2). All subjects received two doses of mRNA vaccine either from Moderna (mRNA-1273) or from Pfizer (BNT162b2) at 4-week or 3-week intervals between doses, respectively (Table S1). Most participants in both cohorts received the Pfizer (BNT162b2) vaccine and there were no significant differences for age, gender, race, ethnicity or vaccine type between the two groups (Table S1a). The responses measured in all assays were not different between the vaccine types. Therefore, all data analyses were conducted irrespective of the specific administered vaccine.

**Fig. 1:**
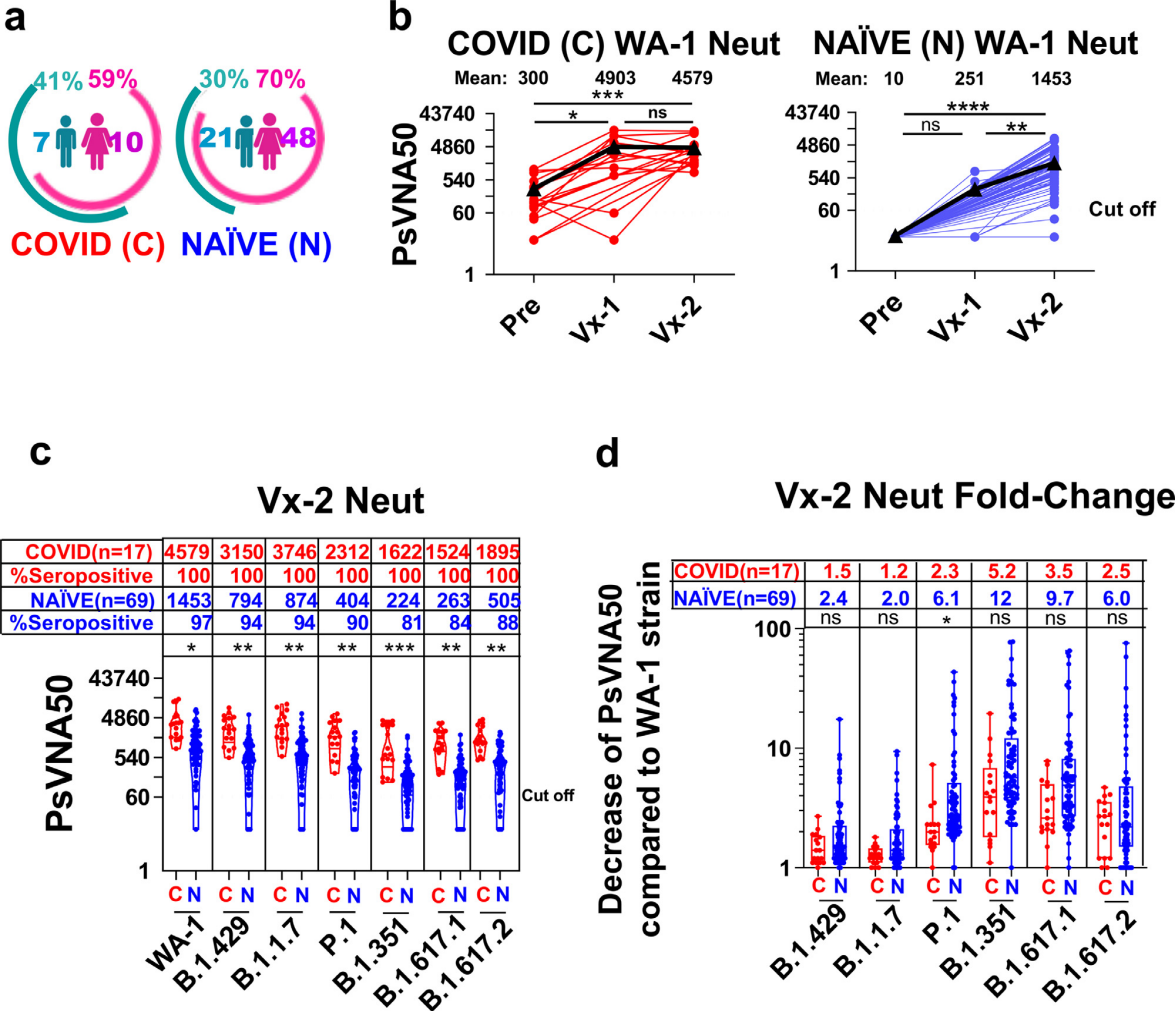
**Neutralizing antibody titers of post-vaccination serum from adults against various SARS-CoV-2 strains**. (a-d) SARS-CoV-2 neutralizing antibody titers in serum of 17 COVID-19 survivors (red) or 69 unexposed naïve (blue) adults as determined by pseudovirus neutralization assay (PsVNA) in 293-ACE2-TMPRSS2 cells with SARS-CoV-2 WA-1 strain, B.1.429 variant, B.1.1.7 variant, P.1 variant, B.1.351 variant, B.1.617.1 or B.1.617.2 variant. PsVNA50 (50% neutralization titer) titers of pre-vaccination (Pre), post-1^st^ (Vx-1) or post-second vaccination (Vx-2) serum samples for COVID survivors (a) and unexposed naïve adults (b) against the vaccine-matched WA-1 strain. Mean PsVNA50 titers values are shown as black triangles and are presented for each vaccination time-point against the SARS-CoV-2 WA-1 on top of the panel. (c-d) Box and whisker plots showing mean values ± SD of PsVNA50 neutralization titers with post-2^nd^ vaccination (panel c), against SARS-CoV-2 WA-1 strain, B.1.429, B.1.1.7, P.1, B.1.351, B.1.617.1 or B.1.617.2 for COVID exposed (C; in red) adults and unexposed naïve adults (N; in blue). (d) Fold-change in PsVNA50 (50% neutralization) titers against emerging variant strain B.1.429, B.1.1.7, P.1, B.1.351, B.1.617.1 and B.1.617.2 for post-second mRNA vaccinated serum from COVID-19 survivors (n=17; in red) or naïve adults (n=69; in blue), in comparison with SARS-CoV-2 WA-1 strain. The numbers above the group shows the median fold-change for each variant are color coded for each of the group matching the colors in the graph. All PsVNA experiments were performed in duplicate and the researchers performing the assay were blinded to sample identity. The variations for duplicate runs were <6%. The data shown are average values of two experimental runs. The statistical significances between the variants within each cohort were performed using R that controlled for age and BMI. The differences were considered statistically significant with a 95% confidence interval when the p value was less than 0.05. (* p ≤ 0.05, ** p ≤ 0.01, *** p ≤ 0.001, **** p≤ 0.0001).

The 17 convalescent seropositive individuals had confirmed SARS-CoV-2 infection between March – November 2020, of these, 13/17 presented with symptomatic PCR-confirmed SARS-CoV-2 infection, while 4 individuals (2 males and 2 females) were asymptomatic but were seropositive prior to vaccination. (Table S1b). While there was an imbalance in the number of participants and the BMI in the COVID-19 exposed *vs*. naïve participants, the age distribution, was similar between the two groups (Table S1a-b). The distribution of male *vs* female participants is shown in Fig. 1a. Male and female cohorts in the naïve and convalescent groups were similar in terms of age, race, ethnicity, BMI, and type of vaccine received (Table S1a). Vaccinations took place between January and February 2021. Samples were collected two weeks after the second vaccination and in 7 naïve and 10 convalescent individuals also after the first vaccination. Since age and BMI can be biologically plausible confounders, data were analyzed for statistical significance amongst cohorts to control for age and BMI as covariates (predictor variables) using a multivariate linear regression model.

The Pseudovirus neutralization assay (PsVNA) was performed using 293-ACE2-TMPRSS2 cell line as previously described [26,30]. A PsVNA50 titer above 1:60 was used as a seropositive cut-off based on current understanding of neutralizing antibody as correlate of protection against COVID-19[31]. For COVID-19 convalescent individuals, pre-vaccination PsVNA50 titers against WA-1 ranged between <1:60 to 1:540 (mean 1:300). Following the first vaccination, 8/10 individuals showed a boost in PsVNA50 titers (mean PsVNA50 of 1:4903), while two individuals showed no increase in titers (Fig. 1b). Second vaccination in this group either increased or maintained high WA-1 neutralizing titers (mean PsVNA50 of 1:4579) (Fig. 1b). In all naïve individuals, pre-vaccination titers were <1:20 (limit of detection). The post-first vaccination PsVNA50 titers were either negative or low (mean 1:250). For 67/69 subjects in this group, the PsVNA50 titers increased significantly after the second vaccination (mean PsVNA50 titers of 1:1453). However, two naïve subjects showed PsVNA50 titers <1:60 (Fig. 1b). In addition to vaccine-homologous WA-1 strain, we measured virus-neutralizing antibody titers against SARS-CoV-2 VOCs (Table S2, Fig. S1 and Fig. 1 c-d).

As expected, the neutralizing antibodies against VOCs after the first vaccination were lower compared with WA-1, with fold decrease ranging between 1.4-5.5 for the convalescent group and between 1.8-22.1 for the naïve group compared with WA-1 strain (Fig. S1). Importantly, single vaccination of convalescent individuals demonstrated neutralization titers against B.1.351 (mean 1:1831) and B.1.617.1 (mean 1:1234), while no neutralization of these VOCs was observed in the naïve group (Fig. S1). A similar pattern of cross-neutralization of SARS-CoV-2 VOCs/VOIs was observed after the second vaccine dose (Fig. S1a). Seropositivity reached 100% for the convalescent group after the second dose, with narrow range of PsVNA50 titers, compared with 80% responders after first dose. The post-second vaccination mean ± SD PsVNA50 titers against the WA-1 strain were 4579 ± 4028 and ranged between 1524 ± 1082 for B.1.617.1 to 3746 ± 2895 against B.1.1.7, among the six SARS-CoV-2 variants (Fig. 1c vs. Fig. S1c; Table S3). In the naïve group, seropositivity reached 97% against WA-1 after the second dose with lower response rates (ranging between 81-94%) against the variants, with lowest titers against B.1.351 VOC (Fig. S1b and Fig. 1c-d). The PsVNA50 titer (mean ± SD) against WA-1 was 1453 ± 1582 that was significantly lower than for the convalescent group (p=0.0285)**.** Similarly, mean ± SD PsVNA50 titers against the six VOCs/VOIs, ranged between 224 ± 305 (against B.1.351) and 874 ± 1007 (B.1.1.7) were significantly lower for the naïve group compared with convalescent group after second mRNA vaccination **(**p<0.05**)** (Fig. 1c and Table S3). Post-second vaccination, the fold decrease in PsVNA50 titers against the VOCs/VOIs compared with WA-1 strains trended higher for the naïve group vs. convalescent group, but due to large intergroup variability they reached statistical significance only against the P.1 VOC (Fig. 1d). We did not observe any statistically significant age-dependent association with neutralizing titers against WA-1 or VOCs/VOIs in either the convalescence or naïve group (Fig. S2)

### 5.2 Vaccination induced binding antibodies against SARS-CoV-2 RBD and its mutants in convalescent vs naive adults

Steady state equilibrium binding of polyclonal serum from post-second dose vaccinated humans was monitored using SPR against RBD of WA-1 and RBD proteins containing key amino acid mutations K417N (found in B.1.351), N501Y (found in B.1.1.7 and other VOCs) and E484K (found in B.1.351, P.1, and B.1.617.1) (Table S2).

**Fig. 2:**
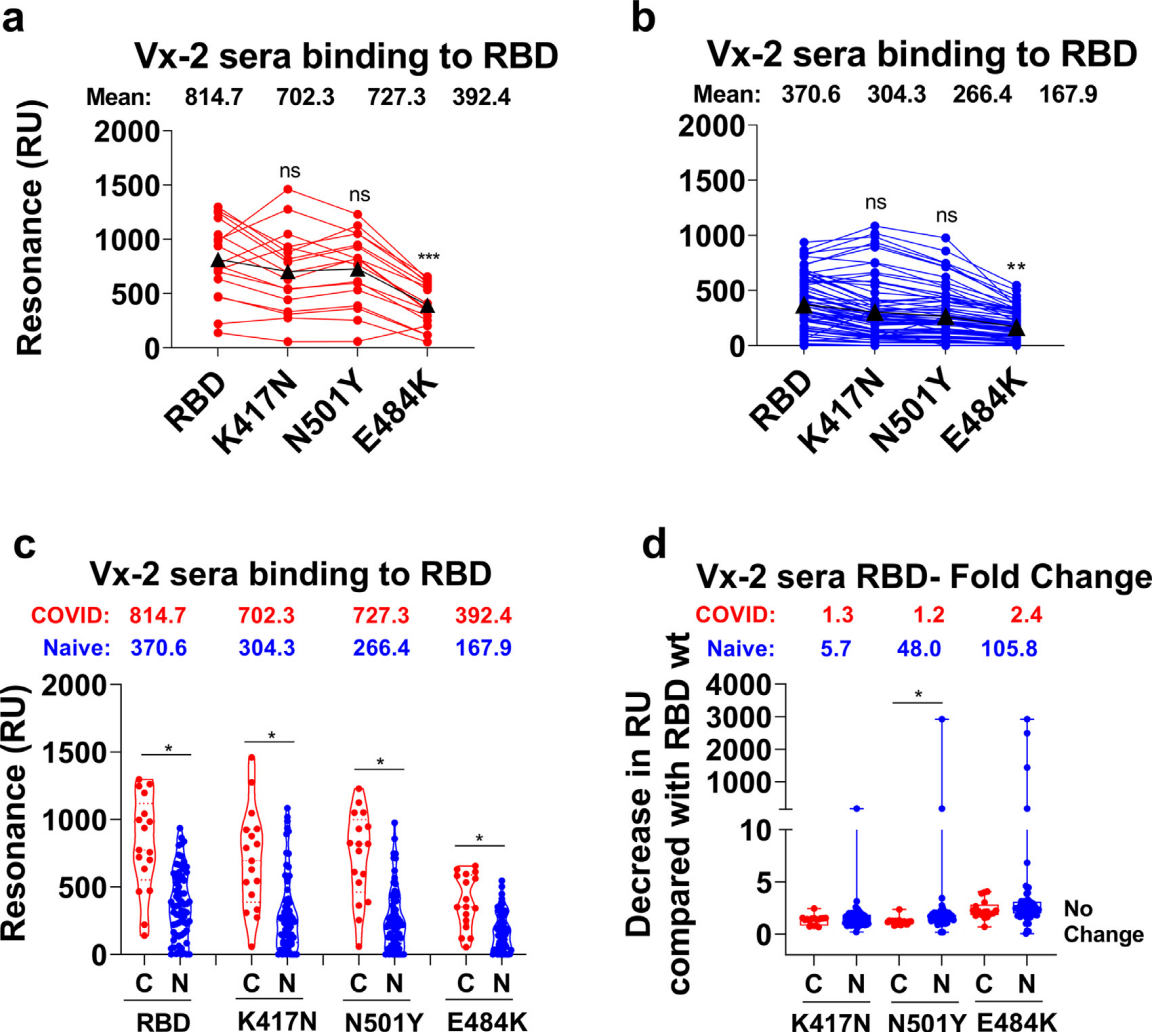
**Binding antibodies in post-second mRNA vaccination serum of COVID-19 survivors vs naïve adults against wild-type SARS-CoV-2 receptor binding domain and its mutants**. (a-b) Total antibody binding (determined by maximum resonance units, Max RU) of 1:10 diluted post-second vaccination serum from COVID-19 survivors (a; n=17) vs naive adults (b; n=69) to purified WA-1 RBD (RBD) and RBD mutants: RBD-K417N, RBD-N501Y and RBD-E484K by SPR. Mean antibody binding values are shown as black triangles and are presented for each RBD. (c) Mean values ± SD of total antibody binding (Max RU) of 10-fold diluted post-2^nd^ vaccination sample with either COVID-19 survivors (n=17; in red; C) vs naive adults (n=69; in blue; N), against purified WA-1 RBD (RBD) and RBD mutants: RBD-K417N, RBD-N501Y and RBD-E484K by SPR. The mean values for Max RU for each RBD are color coded by each group. (d) Fold-decrease in antibody binding to mutants RBD-K417N, RBD-N501Y and RBD-E484K in comparison with RBD from WA-1 strain for post-2^nd^ vaccination samples from COVID-19 survivors (n=17; in red; C) vs naive adults (n=69; in blue; N). The mean values ± SD of fold-change for each mutant is presented and color coded by each group. Line crossing in each panel on Y-axis at scale of 1 denotes no fold-change. All SPR experiments were performed in duplicate, and the researchers performing the assay were blinded to sample identity**.** The variations for duplicate runs of SPR were <5%. The data shown are average values of two experimental runs. The statistical significances between the variants were performed using R that controlled for age and BMI. The differences were considered statistically significant with a 95% confidence interval when the p value was less than 0.05. (*p ≤ 0.05, **p ≤ 0.01, ***p ≤ 0.001, ****p≤ 0.0001).

Following the second mRNA vaccination, the total antibody binding (Max RU) to WA-1 RBD showed diverse range in both groups (Fig. 2a-b). The K417 and N501Y mutations did not significantly impact serum antibody binding to the RBD in either group, but the E484K mutation resulted in a significantly lower antibody binding compared with WA-1 RBD (Fig. 2a-b). The antibody binding (mean ± SD) to RBD (WA-1) or its mutants K417N, N501Y, E484K for the convalescent group were 815 ± 354, 702 ± 370, 727 ± 328, and 392 ± 199, resonance units, respectively. For the naïve group, the mean ± SD RU values were 371 ± 251, 304 ± 281, 266 ± 229, and 168 ± 140, respectively, which is significantly lower than for the convalescent group with p values ranging between 0.0491 and 0.0231 (Fig. 2c and Table S3). The fold decrease in antibody binding between the WA-1 RBD and RBD mutants trended higher for the naïve group (5.7-105.8) compared with the convalescent group (1.3-2.4) (Fig. 2d). These data indicated that the composition of vaccine-induced antibodies in convalescent individuals was different from naïve individuals in terms of relative resistance to individual mutations in the RBD known to affect RBD/ACE2 interactions in VOCs/VOIs.

### 5.3 Vaccination induced antibody affinity maturation in COVID-19 convalescent vs naïve adults to SARS-CoV-2 prefusion spike and RBD

As a surrogate of antibody affinity, antibody off-rate constants, which reflect the stability of the antigen-antibody complex, were determined directly from serially-diluted human polyclonal serum interaction with SARS CoV-2 prefusion spike and RBD using SPR in the dissociation phase only for the sensorgrams with Max RU in the range of 10–150 RU as described before [25], [26], [27] (Fig. S3).

**Fig. 3:**
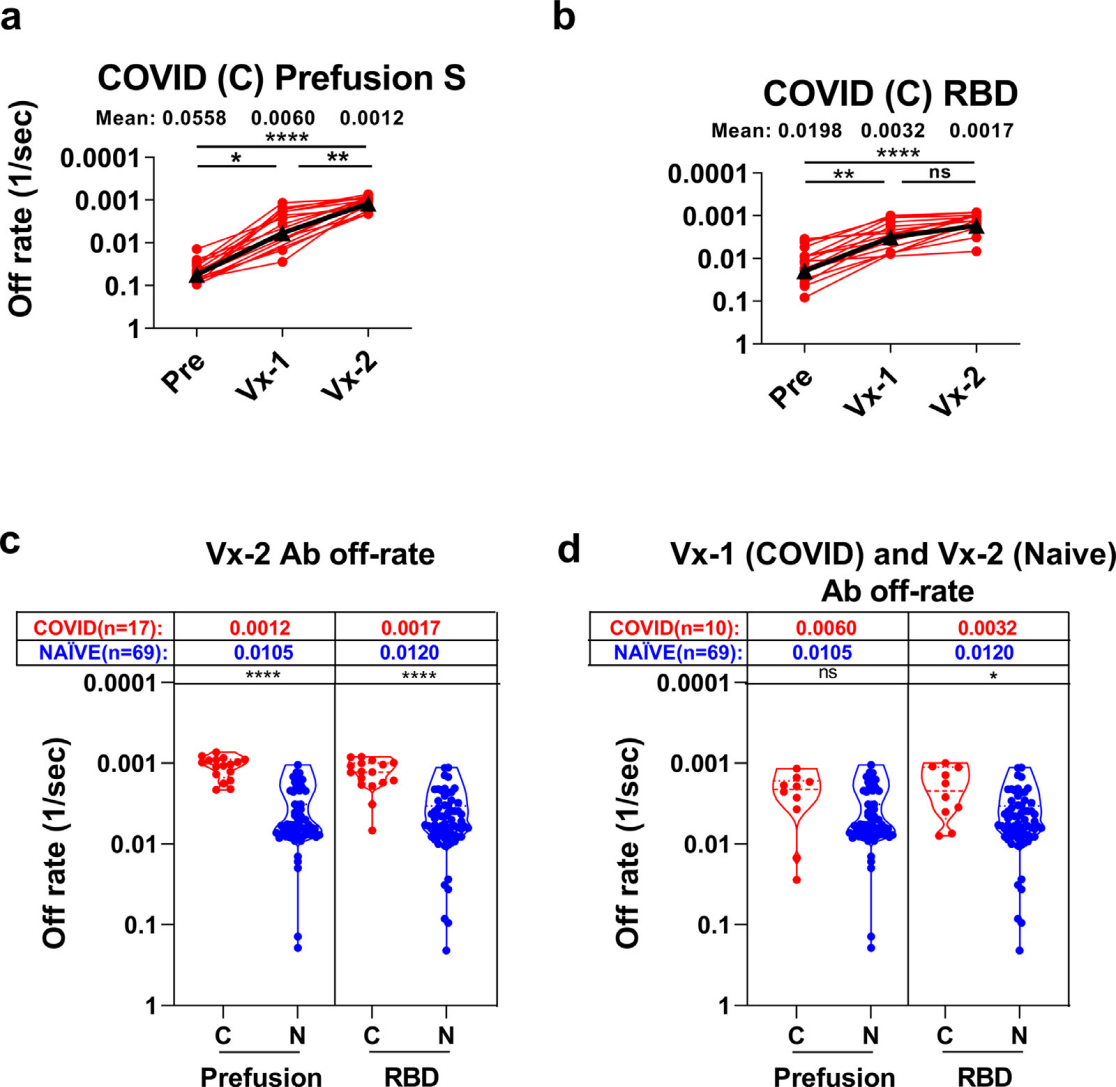
**Antibody affinity maturation of human antibody response following SARS-CoV-2 mRNA vaccination in COVID-19 survivors vs naïve adults.** Polyclonal antibody affinity maturation to SARS-CoV-2 prefusion spike and RBD proteins for pre-vaccination (Pre), post-1^st^ (Vx-1) or post-2^nd^ vaccination (Vx-2) serum samples for COVID exposed adults (n=17; in red) and unexposed naïve (n=69; in blue) adults was determined by SPR. (a-b) Binding affinity of serially diluted post-vaccination serum of each COVID exposed adults to prefusion spike (a) and RBD (b). Antibody off-rate constants that describe the fraction of antibody-antigen complexes decaying per second were determined directly from the serum sample interaction with SARS-CoV-2 proteins using SPR in the dissociation phase as described in Materials and Methods. Off-rate was calculated and shown only for the sample time points that demonstrated a measurable (>5RU) antibody binding in SPR. Antibody affinity of pre- and post-first vaccination serum from naïve unexposed adults were not determined since the prefusion spike or RBD binding antibodies were <5RU for these samples. (c-d) Antibody affinity (as measured by dissociation off-rate per sec) against SARS-CoV-2 prefusion spike and RBD for the post-2^nd^ vaccination samples (panel c) from COVID-19 survivors (n=17; in red; C) vs naïve adults (n=69; in blue; N) or between post-first vaccination serum of COVID-19 survivors (n=17; in red; C) vs post-second vaccination serum from naïve adults (n=69; in blue; N) shown in panel d. The mean values for antibody affinity are color coded by each group. All SPR experiments were performed twice and the researchers performing the assay were blinded to sample identity. The variation for each sample in duplicate SPR runs was <5%. The data shown are the average value of two experimental runs. The statistical significances between the groups or different time-point samples were performed using R that controlled for age and BMI. The differences were considered statistically significant with a 95% confidence interval when the p value was less than 0.05. (*p ≤ 0.05, **p ≤ 0.01, ***p ≤ 0.001, ****p≤ 0.0001).

For the convalescent individuals, the dissociation rates of the pre-vaccination serum antibodies against SARS-CoV-2 prefusion spike (Fig. 3a) or RBD (Fig. 3b) were relatively fast (i.e. low antibody affinity) ranging between 0.1 to 0.01 per sec. Importantly, after the first vaccination, the affinity of the antibodies increased significantly, demonstrating slower dissociation rates from prefusion spike (mean 0.0060/sec) that further significantly affinity matured after the second dose (0.0012 ± 0.0004/sec) (Fig. 3a, Table S3). Similarly, antibody dissociation rates against RBD were slower after the second dose (0.0017 ± 0.0014/sec) compared with the post-first dose (0.0032/sec), but this difference did not reach statistical significance (Fig. 3b).

Naïve individuals did not demonstrate the presence of spike or RBD binding antibodies at pre-vaccination baseline. Following the second vaccination, antibody affinities against either prefusion spike or RBD were 0.0105 ± 0.0280/sec and 0.012 ± 0.0287/sec, respectively. These dissociation rates were significantly faster (∼8-fold) compared with convalescent individuals (p<0.0001) (Fig. 3c and Table S3). Removal of outliers still showed statistically significant difference between convalescent vs naive vaccinees (p values for prefusion was 0.0012; while for RBD, it was 0.003 for group comparison, upon removal of outliers). However, the post-second dose dissociation rates of the naïve individuals against prefusion spike and RBD were similar to those of the post-first vaccination (post-1^st^ dose samples available from 10 convalescent individuals) of the convalescent group (p=0.16 and 0.0383, respectively) (Fig. 3d and Table S3). Removal of outliers resulted in no significant difference between post-first convalescent vs post-second naive vaccinees serum binding antibody affinity to either prefusion spike or RBD (p= 0.118 and 0.2406, respectively). These findings suggest that in convalescent individuals, the first vaccination dose recalls pre-existing memory B cells against SARS-CoV-2 that undergo rapid affinity maturation that continues after the second vaccination. Antibody affinity maturation in the naïve group is delayed compared with the convalescent group but may continue to evolve after vaccination.

### 5.4 Sex differences in SARS-CoV-2 neutralization and antibody affinity maturation following SARS-CoV-2 mRNA vaccination

**Fig. 4:**
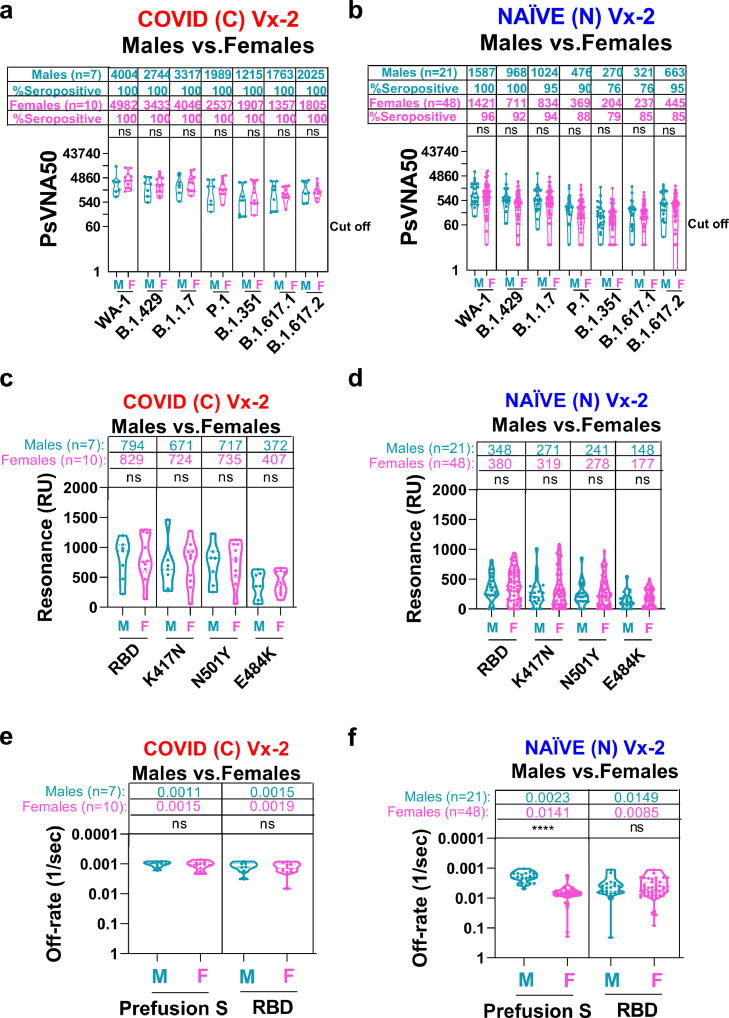
**SARS-CoV-2 neutralization and antibody affinity maturation following SARS-CoV-2 mRNA vaccination in males vs females.** (a-b) Mean values ± SD PsVNA50 neutralization titers of post-2^nd^ vaccination serum from males (in cyan; M) vs. females (in pink; F) against SARS-CoV-2 WA-1 strain, B.1.429, B.1.1.7, P.1, B.1.351, B.1.617.1 and B.1.617.2 belonging to COVID exposed (panel a; n=17) and unexposed naïve (panel b; n=69) group by PsVNA in 293-ACE2-TMPRSS2 cells. Numbers shown are mean PsVNA50 titers for each of the groups. Line crossing in each panel on Y-axis at scale of 60 denotes PsVNA50 cut-off value for seropositivity. (c-d) Antibody binding against SARS-CoV-2 RBD and its mutants following SARS-CoV-2 mRNA vaccination in COVID-19 survivors vs naïve males and females. Total antibody binding (determined by maximum resonance units, RU) of 1:10 diluted post-second vaccination (Vx-2) serum from either males (in cyan) vs. females (in pink) belonging to COVID exposed (n=17; panel c) and unexposed naïve (n=69; panel d) group to purified WA-1 RBD (RBD) and RBD mutants: RBD-K417N, RBD-N501Y and RBD-E484K was determined by SPR. (e-f) Polyclonal antibody affinity to SARS-CoV-2 prefusion spike and RBD proteins for post-2^nd^ vaccination (Vx-2) serum samples from either males (in cyan) vs. females (in pink) belonging to COVID exposed (n=17) and unexposed naïve (n=69) group was determined by SPR. The variation for each sample in duplicate SPR runs was <5% and neutralization assay was <6%. The data shown are the average value of two experimental runs. The mean values are color coded by each group in every panel. The statistical significances between the variants were performed using R that controlled for age and BMI. The differences were considered statistically significant with a 95% confidence interval when the p value was less than 0.05. (*p ≤ 0.05, ****p≤ 0.0001).

The potential impact of sex on immune response to vaccination may influence the long-term effectiveness of vaccination against SARS-CoV-2 in males vs. females. We therefore compared the antibody response of males and females in the convalescent and naïve group after second mRNA vaccination (Fig. 4). The PsVNA50 titers against WA-1 and the six SARS-CoV-2 variants were not significantly different between males vs. females in either convalescent group (p values ranging between 0.21 and 0.9296) (Fig. 4a and Table S3) or in the naïve group (p values ranging between 0.3196 and 0.9869) (Fig. 4b and Table S3). The post-second vaccination binding antibodies to RBD and its mutants were also not significantly different between males vs. females either in the convalescent (p≥ 0.215) **(**Fig. 4c and Table S3) or the naïve group (p values ranging between 0.8117 and 0.9947) (Fig. 4d and Table S3).

On the other hand, when we compared serum antibody affinity against the prefusion spike and RBD proteins after the second vaccine dose, significantly higher antibody affinities were observed in naïve males compared with naïve females, against the prefusion spike (0.0023 ± 0.001 vs 0.0141 ± 0.0331, p <0.0001) (Fig. 4f and Table S3). Removal of outliers still demonstrate statistically significant difference between males vs. females in naive vaccinees against prefusion spike (p value of <0.0001). However, antibody affinity for the RBD did not differ significantly between males vs. females in this naïve group (p >0.9999), upon removal of outliers. This sex-related difference in antibody affinities was not observed in the convalescent group (p= 0.34 and 0.72 for anti-prefusion spike and anti-RBD antibodies, respectively) (Fig. 4e and Table S3). Correlation analysis between antibody affinity with PsVNA50 titers against WA-1 and the VOCs/VOIs, demonstrated a stronger correlation between neutralization titers and antibody affinity against the prefusion spike, with weaker correlation with antibody affinity for the RBD in both males and females (Fig. S4).

## 6 Discussion

Our study revealed both quantitative and qualitative differences in the antibody responses of COVID-19 seropositive convalescent individuals compared with naïve individuals following mRNA vaccination. For both groups, cross neutralization of VOCs was minimally reduced against the B.1.1.7 (Alpha) and B.1.429 (Epsilon), but significantly dropped against the P.1 (Gamma), B.1.617.1 (Kappa), B.1.617.2 (Delta) and B.1.351 (Beta). However, the fold reduction in PsVNA50 titers against Gamma, Kappa, Delta and Beta ranged between 6.2-12 for the post-second vaccination serum from naïve participants compared with only 2.3-5.2-fold reduction for the convalescent vaccine recipients. These finding are in general agreement with several prior studies conducted in similar populations [[19], [20], [21], [22], [23],^32^,^33^].

Antibody binding to RBD proteins with individual mutations revealed significantly reduced binding of post-vaccination serum samples to the RBD with E484K mutation, which is shared between P.1 (Gamma), B.1.351 (Beta), and B.1.617.1 (Kappa) strains. But the decline in antibody binding was ∼102 fold for vaccinated naïve individuals compared with only ∼2.4 reduction for convalescent post-second vaccination samples. In contrast, the K417N had only minimal effect on RBD binding and the N501Y reduced binding only of post-vaccination samples from naïve (∼48 fold) but not from the convalescent group (1.2-fold). Therefore, the neutralization of new circulating variants by vaccine induced antibodies may be impacted by both specific amino acid mutations in the RBD and the specificity/affinity of the polyclonal antibodies that bind to other sites within the SARS-CoV-2 spike. This is further supported by our data demonstrating that antibody affinity against the prefusion spike (more than to RBD) was significantly higher for post-vaccination serum samples from convalescent participants compared with naïve individuals.

Our data along with previous studies suggest that circulating memory B cells with expanded affinity-matured repertoires in convalescent individuals are recalled by vaccination and may enter secondary germinal centers to undergo further affinity maturation [33], [34], [35], [36], [37]. Affinity maturation in the naïve group lags the convalescent group but may continue to evolve in the weeks following post-vaccination. Therefore, in addition to virus neutralization it's important to measure antibody affinity maturation against the entire SARS-CoV-2 spike in order to fully capture the evolution of antibodies after first and second vaccination. In previous studies, we had demonstrated a strong correlation between antibody affinity and protection from challenge with highly pathogenic avian influenza viruses in the ferret model [38,39] and a correlation with lower disease scores and clinical benefit in patients infected with Zika virus[40], Ebola virus [27], influenza virus [41] and COVID-19 [28,29].

We also evaluated the sex differences in the vaccine response of naïve and convalescent individuals. In the convalescent group, no sex differences in any of the quantitative or qualitative antibody measurements were observed. In the naïve group, no significant sex differences were found for the PsVNA50 titers against WA-1 and VOC, in agreement with the prior study [3]. However, unexpectedly, we observed significantly higher affinity of antibodies to the prefusion spike in naïve males compared with females after second vaccination with similar age distribution, a phenomenon that has not been described before, and was not predicted by the neutralization titers measured in these participants. In the current study, there were no significant differences between naïve males and females in race, ethnicity, BMI and vaccine type (Table S1a). The current findings are consistent with our earlier observation following H1N1pdm09 vaccination during the H1N1pdm09 pandemic, where we observed lower antibody affinity in females vs. males against H1N1pdm09 hemagglutinin, even though the post-vaccination HAI titers were similar between the sexes[42].

Limitations of this study include relatively small cohorts of naïve and convalescent individuals with different male/female distribution of 41%/59% vs. 30%/70% in the convalescent vs. naïve cohorts, respectively. In addition, intra group variability of anti-spike affinity was observed among naïve females vs. males. However, even when outliers were removed from the statistical analysis, there was still a strong statistical difference in the anti-prefusion spike binding affinity of naïve males vs. females after the second mRNA vaccine dose (Fig. 4f). Several co-variates (including BMI, race, ethnicity) were comparable between the groups and between males and females in both groups. It is possible that there may be other residual co-variates, which may confound the interpretations of the study. The outcomes of this study merit further evaluation in a large cohort to confirm these findings.

Sokal *et al*, suggested that antibody affinity maturations of the B memory pool in naïve individuals after completing the primary vaccination series continues for at least 6 months[35]. Therefore, it would be important to follow-up the accumulation of mutations and affinity maturation of the memory B cells in males and females to capture long-term antibody evolution and decay. Moreover, a third mRNA vaccine dose (booster) will allow further analysis of the recall responses in males and females in different age groups, and the correlation between antibody affinity and protection against emerging SARS-CoV-2 variants.

This study suggests a differential antibody response in naïve males *vs* females following SARS-CoV-2 vaccination. Therefore, future studies should investigate the potential sex difference in antibody response especially antibody affinity maturation and durability of high-affinity antibodies following SARS-CoV-2 vaccination and the correlation between antibody affinity and vaccine efficacy against emerging SARS-CoV-2 variants that can provide protection against COVID-19.

## Contributors

All authors read and approved the final version of the manuscript.

**Designed research:** S.K. and T.R.

**Performed research:** J.T., G.G., Y.L., C.H., S.R. and S.K.

**Collected clinical samples and provided clinical data**: D.F. and T.R. T. R. verified the underlying data.

**Contributed to Writing:** H.G. and S.K. S.K. and H.G. verified the underlying data.

## Data sharing

All data needed to evaluate the conclusions in the paper are present in the paper and/or the Supplementary Materials.

## Declaration of Competing Interest

The authors whose names are listed immediately below certify that they have NO affi liations with or involvement in anyorganization or entity with any fi nancial interest (such as honoraria; educational grants; participation in speakers’ bureaus;membership, employment, consultancies, stock ownership, or other equity interest; and expert testimony or patent-licensingarrangements), or non-fi nancial interest (such as personal or professional relationships, affi liations, knowledge or beliefs) inthe subject matter or materials discussed in this manuscript.

## Appendix Supplementary materials

Image, application 1

Image, application 2
