# Supplementary material for: Antibody affinity maturation and cross-variant activity following SARS-CoV-2 mRNA vaccination: Impact of prior exposure and sex
Source: eBioMedicine. 2021 Dec 10;74:103748. doi: 10.1016/j.ebiom.2021.103748 (PMC8662368; doi:10.1016/j.ebiom.2021.103748)
Supplement: Supplementary file 1 [file mmc1.pdf]

Supplementary Table S1a: Participant demographics and characteristics

Participant Demographics and Vaccination Type	Convalescent COVID-19 n (%)	Naïve n (%)	Significance p value
Male	7 (41.2)	21 (30.4)	0.9975
Age Mean (Range) in years	38 (22-65)	43.9 (20-73)	0.1125
Vaccine Type, Pfizer	15(88.2)	62 (89.9)	>0.999
Vaccine Type, Moderna	2(11.8)	7 (10.1)	>0.999
BMI Mean	33.72	29.56	**0.0041**
Ethnicity (Non-Hispanic) *	16 (94.11)	67 (97.1)	0.548
Race (White) *	16 (94.11)	69 (100)	0.3

	Convalescent COVID-19 Males n (%)	Convalescent COVID-19 Females n (%)	Significance p value
Age Mean (Range) in years	31.71(24-44)	42.4 (22-65)	0.1125
Vaccine Type, Pfizer	6 (85.7)	9 (90)	0.787
Vaccine Type, Moderna	1 (14.2)	1 (10)	0.787
BMI Mean	30.98	35.65	0.3395
Ethnicity (Non-Hispanic) *	7 (100)	9 (90)	0.866
Race (White) *	7 (100)	9 (90)	0.866

	Naïve Males n (%)	Naïve Females n (%)	Significance p value
Age Mean (Range) in years	43.4 (28-61)	44.08 (20-73)	0.65
Vaccine Type, Pfizer	18 (85.7)	44 (91.6)	0.451
Vaccine Type, Moderna	3 (14.3)	4 (8.3)	0.451
BMI Mean	28.65	28.25	0.794
Ethnicity (Non-Hispanic) *	21 (100)	46 (95.8)	0.911
Race (White) *	21 (100)	48 (100)	>0.99

* Chi square test for independence of variables such as Ethnicity and Race

Supplementary Table S1B: Demographic and vaccination information of COVID exposed and unexposed naïve adults.

COVID convalescent Sample Number	Vaccine Type	Age	Sex	Race	Ethnicity	BMI
1	Moderna	24	M	White	Not Hispanic or Latino	29.1
2	Pfizer	29	M	White	Not Hispanic or Latino	28.7
3	Moderna	40	F	White	Not Hispanic or Latino	46.1
4	Pfizer	22	F	White	Not Hispanic or Latino	26.1
5	Pfizer	25	F	White	Not Hispanic or Latino	34.4
6	Pfizer	59	F	White	Hispanic or Latino	30.4
7	Pfizer	65	F	White	Not Hispanic or Latino	28.8
8	Pfizer	44	M	White	Not Hispanic or Latino	26.5
9	Pfizer	62	F	White	Not Hispanic or Latino	37.5
10	Pfizer	29	M	White	Not Hispanic or Latino	26.5
11	Pfizer	33	M	White	Not Hispanic or Latino	31.4
12	Pfizer	45	F	Black	Not Hispanic or Latino	60.7
13	Pfizer	34	M	White	Not Hispanic or Latino	40.5
14	Pfizer	42	F	White	Not Hispanic or Latino	27.5
15	Pfizer	24	F	White	Not Hispanic or Latino	21.5
16	Pfizer	40	F	White	Not Hispanic or Latino	43.5
17	Pfizer	29	M	White	Not Hispanic or Latino	34.2

SupplementaryTable S1B continued: Demographic and vaccination information of naïve adults.

Naïve Adults Sample Number	Vaccine Type	Age	Sex	Race	Ethnicity	BMI
1	Moderna	23	F	White	Hispanic or Latino	28.5
2	Pfizer	42	F	White	Hispanic or Latino	30.1
3	Moderna	40	M	White	Not Hispanic or Latino	28.5
4	Moderna	20	F	White	Not Hispanic or Latino	20.7
5	Pfizer	73	F	White	Not Hispanic or Latino	29.1
6	Pfizer	28	F	White	Not Hispanic or Latino	26
7	Moderna	38	M	White	Not Hispanic or Latino	24.6
8	Moderna	58	M	White	Not Hispanic or Latino	27.8
9	Moderna	31	F	White	Not Hispanic or Latino	32.4
10	Pfizer	51	F	White	Not Hispanic or Latino	27.6
11	Pfizer	38	M	White	Not Hispanic or Latino	31.2
12	Pfizer	61	F	White	Not Hispanic or Latino	42.3
13	Pfizer	50	M	White	Not Hispanic or Latino	24.3
14	Pfizer	32	M	White	Not Hispanic or Latino	26
15	Pfizer	44	F	White	Not Hispanic or Latino	24.2
16	Pfizer	62	F	White	Not Hispanic or Latino	31.4
17	Pfizer	42	F	White	Not Hispanic or Latino	24.4
18	Pfizer	41	F	White	Not Hispanic or Latino	27.9
19	Pfizer	52	F	White	Not Hispanic or Latino	24.9
20	Pfizer	60	F	White	Not Hispanic or Latino	29.7
21	Pfizer	41	F	White	Not Hispanic or Latino	35.5
22	Pfizer	49	F	White	Not Hispanic or Latino	29.3
23	Pfizer	61	M	White	Not Hispanic or Latino	35.6
24	Pfizer	45	F	White	Not Hispanic or Latino	26.3
25	Pfizer	31	F	White	Not Hispanic or Latino	25.9
26	Pfizer	50	M	White	Not Hispanic or Latino	30.8
27	Pfizer	49	F	White	Not Hispanic or Latino	22.2
28	Pfizer	30	M	White	Not Hispanic or Latino	29.4
29	Pfizer	65	F	White	Not Hispanic or Latino	28.9
30	Pfizer	39	M	White	Not Hispanic or Latino	28.3
31	Pfizer	49	F	White	Not Hispanic or Latino	42.1
32	Pfizer	61	F	White	Not Hispanic or Latino	44.4
33	Pfizer	57	F	White	Not Hispanic or Latino	26.8
34	Pfizer	67	F	White	Not Hispanic or Latino	31.9
35	Pfizer	50	F	White	Not Hispanic or Latino	23.2
36	Pfizer	56	M	White	Not Hispanic or Latino	35.2
37	Pfizer	38	F	White	Not Hispanic or Latino	43.5
38	Pfizer	45	F	White	Not Hispanic or Latino	21.9
39	Pfizer	35	F	White	Not Hispanic or Latino	30.3
40	Pfizer	47	F	White	Not Hispanic or Latino	26.9
41	Pfizer	62	F	White	Not Hispanic or Latino	27.7
42	Pfizer	33	M	White	Not Hispanic or Latino	40.5
43	Pfizer	30	F	White	Not Hispanic or Latino	26
44	Pfizer	28	M	White	Not Hispanic or Latino	26.5
45	Pfizer	49	F	White	Not Hispanic or Latino	20.4
46	Pfizer	40	F	White	Not Hispanic or Latino	26
47	Pfizer	24	F	White	Not Hispanic or Latino	19.6

48	Pfizer	43	M	White	Not Hispanic or Latino	27.2
49	Pfizer	41	F	White	Not Hispanic or Latino	23.3
50	Pfizer	57	F	White	Not Hispanic or Latino	21.2
51	Pfizer	59	M	White	Not Hispanic or Latino	28.5
52	Pfizer	37	M	White	Not Hispanic or Latino	32.7
53	Pfizer	30	F	White	Not Hispanic or Latino	20.1
54	Pfizer	43	F	White	Not Hispanic or Latino	31.7
55	Pfizer	32	F	White	Not Hispanic or Latino	27.8
56	Pfizer	57	M	White	Not Hispanic or Latino	22.7
57	Pfizer	36	F	White	Not Hispanic or Latino	21.4
58	Pfizer	47	F	White	Not Hispanic or Latino	28.2
59	Pfizer	39	M	White	Not Hispanic or Latino	21.1
60	Pfizer	44	F	White	Not Hispanic or Latino	24.1
61	Pfizer	39	F	White	Not Hispanic or Latino	29.1
62	Pfizer	46	M	White	Not Hispanic or Latino	21.4
63	Pfizer	38	F	White	Not Hispanic or Latino	24.8
64	Pfizer	45	F	White	Not Hispanic or Latino	27.8
65	Pfizer	44	M	White	Not Hispanic or Latino	34.4
66	Pfizer	35	M	White	Not Hispanic or Latino	25.1
67	Pfizer	40	F	White	Not Hispanic or Latino	40.8
68	Pfizer	26	F	White	Not Hispanic or Latino	34.1
69	Moderna	34	F	White	Not Hispanic or Latino	23.9

Supplementary Table 2: SARS-CoV-2 variant strain mutations introduced in the spike plasmid for production of SARS-CoV-2 pseudovirions for PsVNA.

SARS-CoV-2 variant	Mutations constructed in the spike plasmids
**B.1.1.7**	H69-V70del, Y144del, N501Y, A570D, D614G, P681H, T716I, S982A, and D1118H
**B.1.429**	S13I, W152C, L452R, D614G
**B.1.526**	L5F, T95I, D253G, E484K or S477N, D614G, A701V
**P.1**	L18F, T20N, P26S, D138Y, R190S, K417T, E484K, N501Y, H655Y, T1027I, D614G, V1176F
**B.1.351**	L18F, D80A, D215G, L242-244del, R246I, K417N, E484K, N501Y, D614G, and A701V
**B.1.617.1**	T95I, G142D, E154K, L452R, E484Q, D614G, P681R, Q1071H
**B.1.617.2**	T19R,G142D,E156del,F157del,R158G,L452R,T478K,D614G,P681R,D950N

Supplementary Table 3: Mean + Standard deviation with statistical p values for group comparisons in all figures

Figure 1C. PsVNA50 titers mean and p values

Group	WA-1	B.1.429	B.1.1.7	P.1	B.1.351	B.1.617.1	B.1.617.2
	Mean±SD	Mean±SD	Mean±SD	Mean±SD	Mean±SD	Mean±SD	Mean±SD
COVID (n=17)	4579±4028	3150±2252	3746±2895	2312±1752	1622±1562	1524±1082	1895±1297
Naive (n=69)	1453±1582	794±870	874±1007	404±440	224±305	263±299	505±493
p value	0.0285	0.0067	0.0074	0.0010	0.0006	0.0015	0.0028

Figure 2C. Resonance units mean and p values

Group	RBD	K417N	N501Y	E484K
	Mean±SD	Mean±SD	Mean±SD	Mean±SD
COVID (n=17)	815±354	702±370	727±328	392±199
Naive (n=69)	371±251	304±281	266±229	168±140
p value	0.0289	0.0460	0.0231	0.0491

Figure 3C. Off-rate Vx-2 mean and p values

Group	Prefusion	RBD
	Mean±SD	Mean±SD
COVID (n=17)	0.0012±0.0004	0.0017±0.0014
Naive (n=69)	0.0105±0.0280	0.0012±0.0287
p value	<0.0001	<0.0001

Figure 3D. Off-rate Vx-1 (COVID) and Vx-2 (Naïve) mean and p values

Group	Prefusion S	RBD
	Mean±SD	Mean±SD
COVID (n=10)	0.0060±0.0087	0.0032±0.0026
Naive (n=69)	0.0105±0.0280	0.0120±0.0287
p value	0.1600	0.0383

Figure 4A. PsVNA50 titers COVID Vx-2 Males vs. Females mean and p values

Group	WA-1	B.1.429	B.1.1.7	P.1	B.1.351	B.1.617.1	B.1.617.2
	Mean±SD	Mean±SD	Mean±SD	Mean±SD	Mean±SD	Mean±SD	Mean±SD
Males (n=7)	4004±4394	2744±1919	3317±2560	1989±1672	1215±1425	1763±1482	2025±1521
Females (n=10)	4982±3941	3433±2519	4046±3208	2537±1860	1907±1662	1357±737	1805±1193
p value	0.4700	0.4900	0.5500	0.3000	0.2100	0.7100	0.9296

Figure 4B. PsVNA50 titers Naïve Vx-2 Males vs. Females mean and p values

Group	WA-1	B.1.429	B.1.1.7	P.1	B.1.351	B.1.617.1	B.1.617.2
	Mean±SD	Mean±SD	Mean±SD	Mean±SD	Mean±SD	Mean±SD	Mean±SD
Males (n=21)	1587±1653	968±1230	1024±1318	476±502	270±399	321±430	663±625
Females (n=48)	1421±1615	711±654	834±854	369±413	204±256	237±219	445±411
p value	0.4597	0.3196	0.5135	0.3135	0.6000	0.9400	0.9869

Figure 4C. Resonance units COVID Vx-2 Males vs. Females mean and p values

Group	RBD	K417N	N501Y	E484K
	Mean±SD	Mean±SD	Mean±SD	Mean±SD
Males (n=7)	794±347	671±397	717±338	372±222
Females (n=10)	829±377	724±371	735±340	407±193
p value	0.3634	0.3579	0.4270	0.2150

Figure 4D. Resonance units Naive Vx-2 Males vs. Females mean and p values

Group	RBD	K417N	N501Y	E484K
	Mean±SD	Mean±SD	Mean±SD	Mean±SD
Males (n=21)	348±221	271±240	241±202	148±132
Females (n=48)	380±265	319±298	278±241	177±143
p value	0.9184	0.9299	0.9947	0.8117

Figure 4E. Off-rate COVID Vx-2 Males vs. Females mean and p values

Group	Prefusion S	RBD
	Mean±SD	Mean±SD
Males (n=7)	0.0011±0.0003	0.0015±0.0008
Females (n=10)	0.0013±0.0005	0.0019±0.0018
p value	0.3400	0.7200

Figure 4F. Off-rate Naive Vx-2 Males vs. Females mean and p values

Group	Prefusion S	RBD
	Mean±SD	Mean±SD
Males (n=21)	0.0023±0.0010	0.0149±0.0445
Females (n=48)	0.0141±0.0331	0.0085±0.0132
p value	<0.0001	0.0537

Supplementary Table S4. Hedge's 'g' for effect size calculations using 'effsize' in R package

Groups	Hedges g*	Effect size
**Serum antibody off-rates**		
Males Naïve Vx2 vs Female Naïve Vx2 Prefusion	1.93	large
Males Naïve Vx2 vs Female Naïve Vx2 RBD	0.52	medium
Naive Vx2 vs COVID Vx2 Prefusion	1.84	large
Naive Vx2 vs COVID Vx2 RBD	1.57	large
Males COVID Vx2 vs Female COVID Vx2 Prefusion	0.4	small
Males COVID Vx2 vs Female COVID Vx2 RBD	0.23	small
**Serum antibody binding**		
Males Naïve Vx2 vs Female Naïve Vx2 RBD	0.05	small
Males COVID Vx2 vs Female COVID Vx2 RBD	0.02	small
Naive Vx2 vs COVID Vx2 RBD	0.8	large
**Serum antibody neutralization (PsVNA50)**		
Males Naïve Vx2 vs Female Naïve Vx2 Neutralization WA1	0.2	small
Naïve Vx2 vs COVID Vx2 Neutralization WA1	1.24	large
Males COVID Vx2 vs Female COVID Vx2 Neutralization WA1	0.39	small

*Data reported are g values with a 95% confidence interval

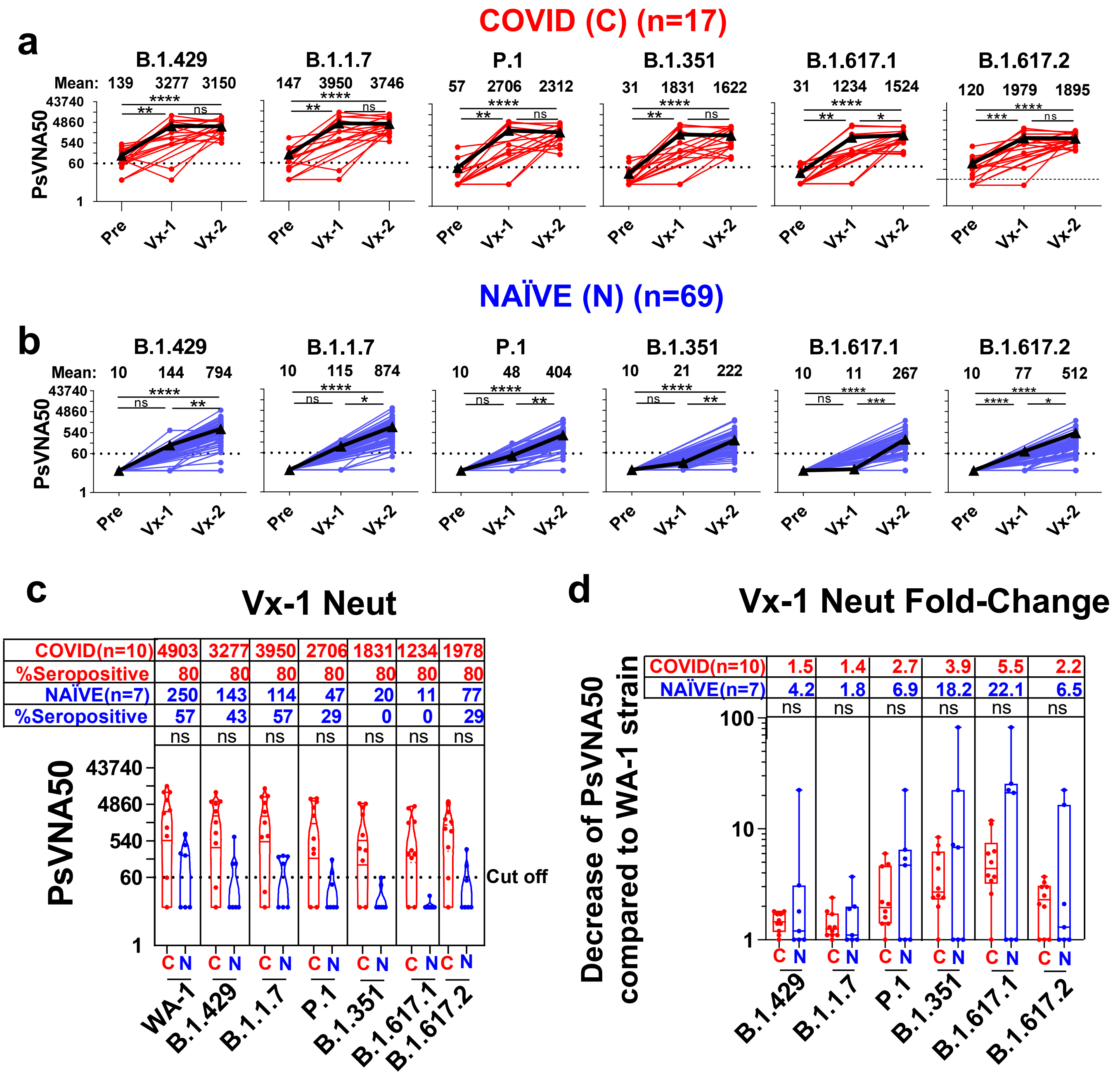

Supplementary Figure 1: Neutralizing antibody titers of post-vaccination serum from adults against various SARS-CoV-2 strains.

SARS-CoV-2 neutralizing antibody titers in serum of 17 COVID-19 survivors (red) or 69 unexposed naïve (blue) adults as determined by pseudovirus neutralization assay(PsVNA) in 293-ACE2-TMPRSS2 cells with SARS-CoV-2 WA-1 strain, B.1.429 variant, B.1.1.7 variant, P.1 variant, B.1.351 variant, B.1.617.1 or B.1.617.2 variant. PsVNA50 (50% neutralization titer) titers of pre-vaccination (Pre), post-1st (Vx-1) or post-2nd vaccination (Vx-2) serum samples for COVID survivors (a) and unexposed naïve adults (b) against the vaccine-matched WA-1 strain. Mean PsVNA50 titers values are shown as black triangles and are presented for each vaccination time-point against the SARS-CoV-2 WA-1 on top of the panel.. Differences among various time-points were performed using R. The differences were considered statistically significant with a 95% confidence interval when the p value was less than 0.05. (* p ≤0.05, ** p ≤0.01, *** p ≤ 0.001). (c) Box and whisker plots showing mean values + range of PsVNA50 neutralization titers with post-1st vaccination against SARS-CoV-2 WA-1 strain, B.1.429, B.1.1.7, P.1, B.1.351, B.1.617.1 or B.1.617.2 for COVID exposed (C; in red) adults and unexposed naïve adults (N; in blue). (d) Fold-change in PsVNA50 (50% neutralization) titers against emerging variant strain B.1.429, B.1.1.7, P.1, B.1.351, B.1.617.1 and B.1.617.2 of post-first mRNA vaccination serum from COVID-19 survivors (n=10; in red) or naïve adults (n=7; in blue) in comparison with SARS-CoV-2 WA-1 strain. Data shown is mean value + range for fold-change in PsVNA50 titers for each of the groups. Line crossing in each panel on Y-axis at scale of 1 denotes no fold-change.

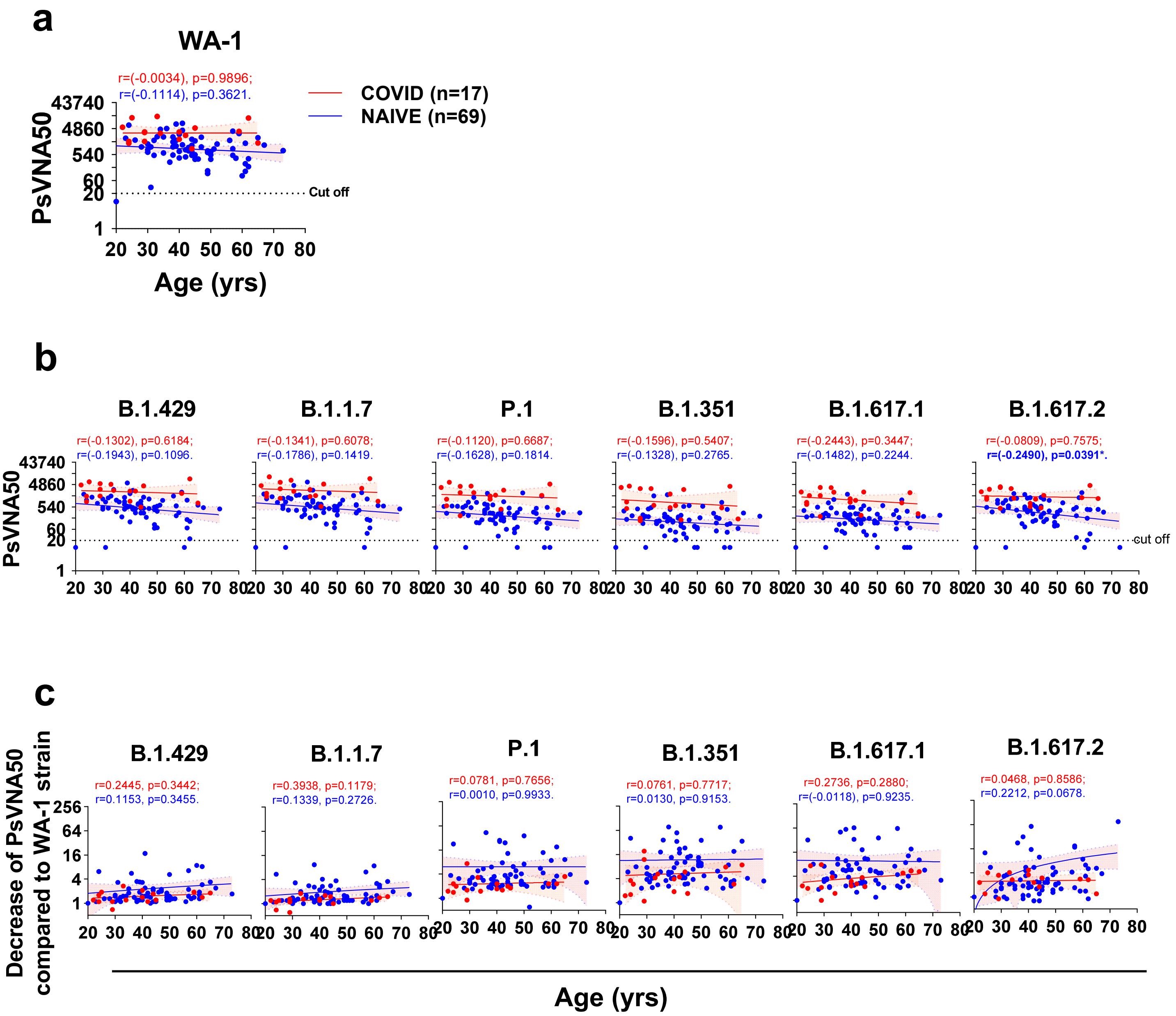

Supplementary Figure 2: Relationship of post-2nd vaccination serum neutralizing antibodies in COVID survivors and naïve adults with age. Correlation analysis between age and serum neutralization antibody levels of COVID exposed (n=17; in red) and unexposed naïve (n=69; in blue) group against SARS-CoV-2 WA-1 strain (a) or SARS-CoV-2 variants (b) measured by pseudovirus neutralization assay in 293-ACE2-TMPRSS2 cells. Dotted line indicate the neutralization titer cutoff (PsVNA50 of 60) for seropositive determination. (c) Correlation analysis between age of the participants and fold-decrease in PsVNA50 neutralization titers against emerging VOC strain B.1.429, B.1.1.7, P.1, B.1.351 and B.1.617.1 for post-second mRNA vaccinated serum from COVID-19 survivors (n=17; in red) or naïve adults (n=69; in blue), in comparison with SARS-CoV-2 WA-1 strain. Correlation analysis was performed using linear regression model and associated Pearson's correlation coefficients (r) and linear regression significance (p) are shown; shading represents 95% CI.

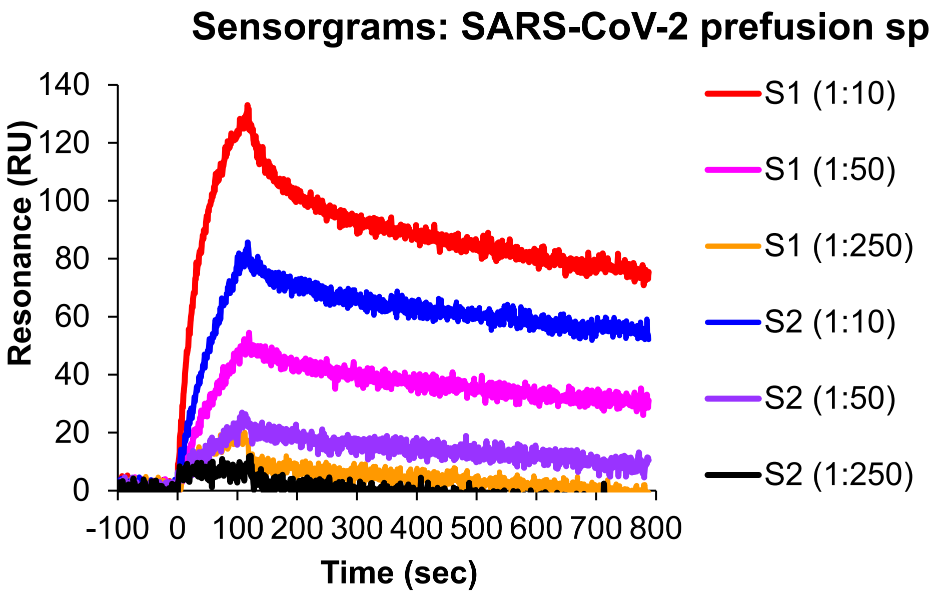

Supplementary Figure 3: Steady-state equilibrium analysis of post-vaccination antibodies binding by SPR. Related to figure 3. Serial dilutions of post-vaccination samples were injected simultaneously SARS-CoV-2 prefusion spike ectodomain captured on a Ni-NTA sensor chip and on a surface free of protein (used as a blank). Binding responses from the protein surface were corrected for the response from the mock surface and for responses from a separate, buffer only injection. Unvaccinated control sample at 10-fold dilution did not show any binding in SPR. Antibody off-rate constants, which describe the fraction of antigen-antibody complexes that decay per second, were determined directly from the serum sample interaction with prefusion spike using SPR in the dissociation phase only for the sensorgrams with Max RU in the range of 10-100 RU and calculated using the BioRad ProteOn manager software for the heterogeneous sample model.

Supplementary Figure 4

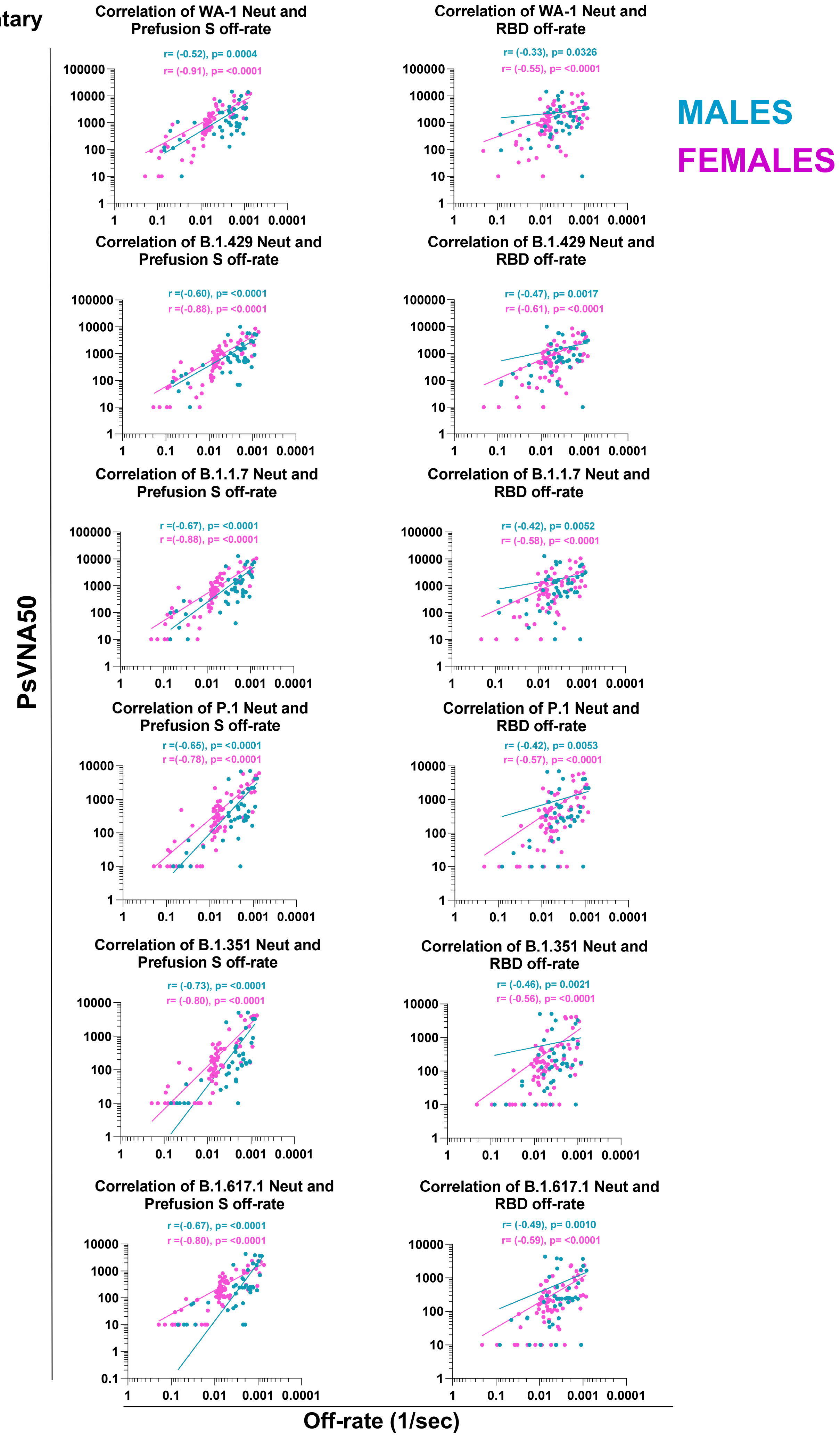

Supplementary Figure 4: Relationship of post-vaccination SARS-CoV-2 serum neutralizing antibodies in COVID convalescent and naïve adults with antibody affinity against SARS-CoV-2 prefusion spike or RBD. Correlation analysis between serum PsVNA50 neutralization antibody titers generated following first and second vaccination of COVID exposed (n=17) and unexposed naïve (n=69) adults against vaccine-matched SARS-CoV-2 WA-1 and emerging SARS-CoV-2 VOCs B.1.429, B.1.1.7, P.1, B.1.351 and B.1.617.1, and antibody affinity against either SARS-CoV-2 prefusion spike (a) or RBD (b) of males (cyan) vs females (pink). Correlation analysis was performed using non-linear regression model and associated Spearman's correlation coefficients (r) and regression significance (p) are shown.
