# Supplementary material for: Antibody affinity maturation and cross-variant activity following SARS-CoV-2 mRNA vaccination: Impact of prior exposure and sex
Source: eBioMedicine. 2021 Dec 10;74:103748. doi: 10.1016/j.ebiom.2021.103748 (PMC8662368; doi:10.1016/j.ebiom.2021.103748)
Supplement: Supplementary file 2 [file mmc2.docx]

## Supplementary Materials:

## Supplementary Table 1: Demographic and vaccination information of COVID exposed and unexposed naïve adults.

Supplementary Table 2: SARS-CoV-2 variant strain mutations introduced in the spike plasmid for production of SARS-CoV-2 pseudovirions for PsVNA.

Supplementary Table 3: Mean + standard deviation with statistical p values for group comparisons in all figures.

Supplementary Table 4: Hedge's 'g' for effect size calculations using 'effsize' in R package

Supplementary Figure 1: Neutralizing antibody titers of post-vaccination serum from convalescent or naïve adults against various SARS-CoV-2 strains.

Supplementary Figure 2: Relationship of post-second vaccination serum neutralizing antibodies in COVID survivors and naïve adults with age.

Supplementary Figure 3: Steady-state equilibrium analysis of post-vaccination antibodies binding by SPR.

Supplementary Figure 4: Relationship of post-vaccination SARS-CoV-2 serum neutralizing antibodies in COVID convalescent and naïve males and females with antibody affinity against SARS-CoV-2 prefusion spike or RBD.
